# Examining the association between men's gender equitable attitudes and contraceptive outcomes in rural Maharashtra, India

**DOI:** 10.1016/j.dialog.2024.100168

**Published:** 2024-01-20

**Authors:** Mohan Ghule, Anvita Dixit, Nicole E. Johns, Madhusudana Battala, Shahina Begum, Sarah Averbach, Jay G. Silverman, Niranjan Saggurti, Anita Raj

**Affiliations:** a Center on Gender Equity and Health, Division of Infectious Diseases and Global Public Health, School of Medicine, University of California San Diego, San Diego, USA; b Joint Doctoral Program in Public Health (Global Health track), University of California San Diego/San Diego State University, USA; c Population Council, New Delhi, India; d ICMR-National Institute for Research in Reproductive and Child Health, Mumbai, India; e Department of Obstetrics, Gynecology, and Reproductive Sciences, School of Medicine, University of California San Diego, USA; f Newcomb Institute, Tulane University, New Orleans, LA, USA

**Keywords:** Gender attitudes, Family planning, Contraceptive communication, Male engagement, Gender-equitable men scale

## Abstract

**Background:**

Previous literature suggests that men reporting more gender-equitable attitudes are more likely to use condoms, but there is a paucity of data evaluating whether these attitudes are associated with contraceptive communication and use. The objective of this study is to test the hypothesis that men reporting more gender-equitable attitudes will be more likely to (a) engage in contraceptive communication with their wives and (b) that they and/or their wives will be more likely to use all forms of family planning, compared to men with less equitable attitudes.

**Methods:**

Using cross-sectional dyadic survey data from young married couples from rural Maharashtra, India (*N* = 989), we assessed the associations between men's gender role attitudes and a) spousal contraceptive communication and b) contraceptive use by type (none, traditional, condoms, pills, or IUD). The contraceptive use outcome is based on wives' report. We assessed these associations via bivariate *t*-test (communication outcome) or ANOVA test (contraceptive type outcome), as well as unadjusted and adjusted logistic (communication outcome) and multinomial logistic (contraceptive type outcome) regression models. Adjusted models included sociodemographic factors selected a priori based on established associations with gender-equitable attitudes and/or our assessed outcomes.

**Findings:**

Men with more gender-equitable attitudes were more likely to discuss family planning with their wives (AOR = 1·05, 95%CI 1·03-1·07, *p* < 0·001) and to use condoms (ARRR = 1·03, 95%CI 1·00-1·06, *p* = 0·07). There was no association between gender-equitable attitudes and use of other types of contraception.

**Interpretation:**

While gender-equitable attitudes among men may facilitate condom use and family planning communication in marriage, they do not appear to be linked with greater likelihood of use of more effective types of contraceptive use. This suggests that males supportive of gender equity may take greater responsibility for family planning vis a vis a less effective contraceptive, condoms, in the absence of more effective short-acting contraceptives for men.

**Funding:**

The 10.13039/100000002National Institutes of Health [Grant number 5R01HD084453-01A1] and the 10.13039/100000865Bill & Melinda Gates Foundation, Seattle, WA [grant number INV-002967].

## Research in context

### Evidence before this study

Researchers have long recognized the importance of engaging men in family planning, in ways that are supportive and respectful of their female partners. Interventions which aim to improve gender-related norms and attitudes are one key strategy for male engagement, with the assumption that more equitable norms will lead to greater support for contraceptive communication and use. We searched PubMed for peer-reviewed articles reporting on ‘masculinity’ or ‘gender’ ‘norms’ or ‘attitudes’ and ‘contraceptive use’ or ‘contraceptive communication’ (and related key terms). We found extensive research from the field of HIV indicating that greater masculinity norms and attitudes are associated with lower male condom use, and that gender-transformative interventions that target restrictive masculinity norms are effective in increasing male condom use. Prior studies from India have also shown that men's attitudes about gender equity are positively associated with condom use to prevent HIV. However, there was little research examining associations between men's reported gender attitudes and other types of contraceptives, condom use for contraceptive reasons, or contraceptive communication with partners.

### Added value of this study

This is the first study to our knowledge to examine men's gender equitable attitudes and their association with contraceptive use (for many times of contraception) and contraceptive communication with their partners. We find that more gender equitable attitudes were associated with greater condom use and greater contraceptive communication, but found no association with other forms of contraceptive use.

### Implications of all the available evidence:

Our study, along with prior evidence, suggests that men's gender-related attitudes are important predictors of condom use. Our study expands upon prior research to also demonstrate and association between these attitudes and contraceptive communication, and to demonstrate a null relationship between gender equitable attitudes and other use of other forms of contraception. These findings suggest that incorporation of gender equity considerations, and the use of gender-transformative intervention approaches, are valuable for increasing contraceptive utilization but not alone sufficient to enourage increased uptake of a range of effective methods.

## 1 Introduction

Family planning supports women's health and development [1] and has been part of the public health system in India since 1952 [2], but use of modern contraception for birth spacing remains very low due to persistent early in marriage fertility norms and ongoing son preference in the country [2,3]. In the past five years, government efforts have sought to improve uptake of modern contraceptives early in marriage and to improve pregnancy planning and inter-pregnancy spacing, but without clear guidance on gender equity issues [4], which can be a key impediment in contexts such as rural India where contraceptive use remains relatively low and traditional gender norms are pervasive [2,5]. Prior research shows that interventions that disrupt gender norms improve contraceptive uptake among young married couples across a range of settings [6,7], but few evaluated programs include male involvement in their approach [6,8,9]. Research from across national contexts documents the importance of male support for family planning to improve contraceptive uptake among couples and the value of gender transformative programming for men to engender this support [[10], [11], [12], [13]]. However, in practice, such effects can be difficult to achieve [14]. More research on gender roles and contraceptive use among men can help guide this programming. This study seeks to examine the association between men's gender norm ideologies and contraceptive behavior outcomes among young husbands in the context of rural India, where spacing contraceptive use and male engagement in family planning remain low [[15], [16], [17], [18]].

Consideration of men's gender ideologies related to family planning aligns with the Can-Act-Resist framework for understanding women's agency in health as part of an empowerment process, positing that women's agency in family planning would be facilitated by male support for women's choice and self-determination in reproductive practices, and would require gender normative shifts related to male entitlement to control women's fertility [19]. Prior research from India assessing gender roles and contraceptive use has focused on men's attitudes towards acceptability of marital violence as an indicator of men's masculinity ideology. These studies show significant associations between less accepting attitudes towards marital violence (e.g. lower masculinity ideology) and greater marital contraception use at all, reversible contraceptive use specifically, and smaller desired family size [[20], [21], [22]]. A stronger and more comprehensive measure of masculinity ideology is the Gender Equitable Men Scale (GEMS), which assesses men's normative beliefs related to male gender roles, male sexuality, and male dominance over women [[23], [24], [25], [26]]. This measure, originally designed to understand effects of masculinity ideologies on sexual and HIV risk-taking behaviors, has been tested cross-nationally, including in India and South Asia, demonstrates strong validity and reliability with diverse populations, and documents significant associations with men's reports of IPV perpetration, number of sex partners, and condom use [23,24,[26], [27], [28]]. Research from India and South Asia using the GEMS measure also documents associations between more traditional (less gender equitable) norms and desire for more sons [26,28], with rural men more likely than urban men to report traditional (less gender equitable) norms [26,28]. While son preference is associated with non-use of contraceptives among married couples in India, and both son preference and non-use of contraceptives are more likely in rural compared with urban settings in India [2], none of this prior work has focused on examination of the associations between men's gender ideologies and contraceptive behaviors beyond condom use.

Approaches to FP programming which directly address men's gender norms and attitudes are particularly relevant in contexts such as Maharashtra, India, where reversible contraceptive use remains low and endorsement of harmful gender norms remains common. In Maharashtra, 64% of women use modern contraception, dominated by female sterilization (49% of women) and condom use (10%). Use of other methods is low, and the majority of women report never having had counselling with a FP provider [2,29]. Estimates also show that only 25% of non-sterilized women of childbearing age use modern contraception in the Junnar block of Pune district, the specific area under study [30]. One in six men (16%) in the state endorse the statement that ‘contraception is women's business and a man should not have to worry about it’. [2,29] Though state-level indicators reflective of gender equity are largely better than national averages (for example, lower sex ratio at birth, higher literacy), there are still persistent harmful gender norms, such as those supportive of violence against women [29].

For more than 25 years, researchers have recognized the importance of engaging men in family planning, in ways that are supportive and respectful of their female partners who will have to bear the physical efforts of pregnancy and childbirth [31]. More recent evidence further highlights the growing interest of men as users of contraception, particularly in the form of condom use, and the inadequacy of existing family planning programs in terms of reaching and engaging men effectively [13]. The HIV pandemic heightened examination and engagement of men for condom use; extensive research from the field of HIV has found that greater endorsement of masculinity norms and attitudes are associated with lower likelihood of male condom use, and that gender-transformative interventions that target restrictive masculinity norms are effective in increasing male condom use [32]. As previously discussed, studies have shown that men's attitudes supportive of gender equity are positively associated with condom use, in part for the prevention of HIV [[33], [34], [35]]. However, there is little research examining associations between men's reported masculinity ideologies and contraceptive practices, including FP communication with their partner and use of contraceptives other than condoms.

This study assesses the association between men's gender attitudes and a) spousal communication about contraceptive use and b) use of contraceptives, overall and by specific type, among young married couples in rural Maharashtra, India. We include communication as an indicator of male engagement in family planning. We hypothesize that couples where the husband has more gender-equitable attitudes, compared to those where the husband has less equitable attitudes, are more likely to engage in couple communication about family planning and have a higher probability of use of diverse types of modern contraceptives. These findings can offer important insight into the importance of male gender attitudes, and the community level norms that may reinforce them, as targets for gender-transformative family planning interventions with men.

## 2 Methods

### 2.1 Sample and recruitment

This study utilizes a cross-sectional analysis of baseline survey data from married young couples participating in the CHARM2 [Counselling Husbands and wives to Achieve Reproductive health and Marital equity] family planning evaluation trial, conducted in the rural Junnar district of Maharashtra, India.

CHARM2 is a counselling intervention that aims to increase uptake of contraceptives, prevent unintended pregnancy, and decrease interpersonal violence. Couples who were not currently married or cohabiting in the village for at least the last three months, or who were using a permanent contraceptive method (female or male sterilization), were not eligible to participate in the study. The CHARM2 study is described in greater detail elsewhere [36].

Data were collected from couples who were eligible and recruited into the CHARM2 study, in which the wife was aged 18-29 years, and no infertility or sterilization was indicated for either spouse (*N* = 1201 couples). Participants were recruited from households from 20 geographic clusters, based on primary health centre catchment areas, and were randomized at the cluster level into intervention and control conditions prior to the study. The sample for the current analyses were restricted to those couples in which the women did not self-report current pregnancy (e.g. women who were actively pregnant at time of survey; women who delivered within the prior three months were included) and for which all outcome and independent variable data were available. We thus excluded 212 of 1201 couples, where either wife was pregnant (*n* = 199) or where husband was missing GEMS score (*n* = 9). We utilized a categorical contraceptive use by type outcome variable. No husbands were missing responses for FP discussion with wife. This resulted in a final analytic sample of *N* = 989 couples.

### 2.2 Data collection and procedure

Data was collected between September 2018 and June 2019 by trained field research investigators. In-person gender-matched interviews were carried out using electronic tablets with couples (husband and wife separately) who consented to the interviews. The length of survey was approximately 40 min per interview. Privacy was ensured and maintained throughout the survey interview, which was carried out at the participant's home. Self-report data were collected on socio-demographics, marital history, sexual and reproductive health matters including family planning, gender based violence, and mental health. Data quality and fieldwork were monitored by trained field coordinators and Indian Council of Medical Research/National Institute for Research in Reproductive Health (ICMR-NIRRH) research staff. No monetary incentives were offered for participation.

Ethical approval for this study was obtained from the National Institute for Research in Reproductive Health Ethics Committee (#270/2014, initial approval 11/12/2018), the Population Council Institutional Review Board (#EX2018012, initial approval 7/16/2018), and the University of California San Diego Institutional Review Board (#161797, initial approval 1/19/2017; #190167, initial approval 4/12/2019). Written informed consent was obtained from all respondents at the time of baseline survey. The interviewers documented the receipt of written informed consent on individual consent forms.

### 2.3 Survey measures

#### 2.3.1 Dependent variables

Current contraceptive use was assessed based on women's report of type of contraceptive method used in past three months. Though men were also asked questions of contraceptive method use, female reports were considered more reliable and were less likely to be missing; women may also have used some methods (e.g. pills, IUD) without their male partner's knowledge. Though we did not directly assess recent covert contraceptive use, 3% of women reported covert contraceptive use at some point in their lifetime; cell sizes were too small to allow for analyses on covert use as an outcome. Responses were categorized into five categories: none, traditional (withdrawal/rhythm method), condoms, oral contraceptive pills (OCPs), and intrauterine device (IUD). We excluded from these analyses *n* = 4 women who were using injectable contraception, as the cell size was too small to allow for analysis. For women who reported use of more than one modern method in the prior three months (*n* = 6), they were categorized based on the most effective method used (IUD first, OCP second, condom third). Women who reported both use and discontinuation of contraception in the prior three months (*n* = 14) were classified based on the method used rather than classified as non-users.

Couple's family planning discussion was assessed via a single Yes/No item, which was asked to men as whether they discussed with their wife what to use or do to prevent or stop a pregnancy in the past three months. Though this item was asked of both women and men, male response on dependent variables was utilized as the focus of this paper was on male attitudes, which was asked only of men.

#### 2.3.2 Independent variable

Men's attitudes towards gender norms were assessed using the previously well-established 24-item Gender-Equitable Men Scale (GEMS) (see Appendix Table 1 for full scale) [23,24,37]. The GEMS measure was developed to capture a range of domains inclusive of gender norms, masculinities, sexuality, and violence, and is based on the theory of gender as a social construct, wherein gender is developed through social interactions. The study was initially developed in Brazil, with several proposed country-specific adaptations. We utilize the full 24-item original measure. Items in the scale include: “A man should have the final word about decisions in his home,” “It is important that a father is present in the lives of his children, even if he is no longer with the mother,” “A man needs other women, even if things with his wife are fine,” “Men are always ready to have sex,” and, “I would be outraged if my wife asked me to use a condom.” Responses on a 3-point scale were measured: 1-don't agree, 2-partially agree, and 3-agree (responses were reverse coded for seven indicated items, noted in Appendix Table 1). The scale has been previously validated as a continuous measure in the Indian and other developing country contexts. The reliability analysis for this study population showed a Cronbach's alpha value of 0·70, suggesting that it is a moderately reliable scale for current sample. The items were summed together (range 24-72), with a higher score indicating greater support of gender-equitable norms among men. As noted previously, this measure has been cross-nationally validated in prior research including in the context of India [[25], [26], [27]].

#### 2.3.3 Sociodemographic variables and intimate partner violence

Additional variables included as covariates from survey data were men's age (continuous), women's age (continuous), men's education (none or primary, secondary or more), women's education (none or primary, secondary or more), women's caste (General, Scheduled Caste/Scheduled Tribe/Other Backward Class [note that these are official government designations [2]]), household Below Poverty Line card holder (Yes/No), parity (0, 1, 2+), and women's report of any intimate partner violence including physical, sexual, or emotional in the past 12 months (Yes/No). We also present descriptive statistics on couple age difference, but do not include this in adjusted models due to collinearity with husband and wife ages. All sociodemographic characteristics included in adjusted models - men's and women's age, men's and women's education, women's caste, household Below Poverty Line card holder, and parity - were selected a priori based on upon established associations between the characteristic and FP use and availability in the survey data [2,38,39]. We also controlled for IPV as a potential mediator since it may be associated with both traditional gender norms and contraceptive outcomes [38,39].

### 2.4 Data analysis

Descriptive statistics for GEMS scores were calculated, and bivariate associations with outcomes were tested, using ANOVA for the categorical outcome (Type of FP method use), and *t*-test for the dichotomous outcome (FP discussion). We then conducted bivariate comparisons of outcomes with sociodemographic characteristics, again using ANOVA or *t*-tests as appropriate. We next assessed associations between GEMS score and sociodemographic characteristics utilizing unadjusted linear regression models. We then constructed unadjusted and adjusted multinomial logistic regression models for the categorical current family planning method use outcome, and unadjusted and adjusted binary logistic regressions for the binary family planning discussion outcome. As an additional post-hoc analysis, we also examined the association between GEMS score and IPV. We found no evidence of multicollinearity among a priori included sociodemographic factors in adjusted models. We therefore retained all sociodemographic variables in adjusted models. No further model building was conducted. Odds ratios (ORs) and adjusted odds ratio (AORs) are reported for binary logistic regression models with the binary outcome of FP discussion; relative risk ratios (RRRs) and adjusted relative risk ratios (ARRRs) are reported for multinomial logistic regression models with the categorical outcome of type of FP method used. Significance was set at *p* < 0·05 for all comparisons and 95% confidence intervals (CIs) are reported throughout. All analyses were conducted using STATA 15.1.

## 3 Results

### 3.1 Outcomes and gender equitable attitudes score summary statistics

In this sample, the majority of women (61%, *n* = 604) reported currently using a family planning method, comprised of 23% (*n* = 231) reporting traditional methods (withdrawal and rhythm), 25% (*n* = 252) condoms, 9% (*n* = 89) IUD, 3% (*n* = 32) OCPs (see Table 1). The majority of men (61%, *n* = 605) reported having a discussion with their wives on what to use or do to prevent/stop a pregnancy within the past three months. Mean GEMS score was 54·3 (SD 6·2) in the sample.

**Table 1:** Family planning method use and discussion of family planning frequencies by sociodemographic characteristics of married couples in baseline sample from CHARM2 in rural Maharashtra, India (*N* = 989).

Variable	Overall, n (%)	Type of family planning method use					*p*-value	Discussion on family planning		p-value
		None	Rhythm/Withdrawal	Condoms	Pills	IUD		Yes	No	
*Total (N, %)*	*989 (100)*	*385 (38*·*9)*	*231 (23·4)*	*252 (25*·*5)*	*32 (3·2)*	*89 (9*·*0)*		*605 (61·2)*	*384 (38*·*8)*	
		**Mean (SD)**	**Mean (SD)**	**Mean (SD)**	**Mean (SD)**	**Mean (SD)**		**Mean (SD)**	**Mean (SD)**	
GEMS score	54·32 (6·17)	54·02 (6·17)	54·28 (6·25)	55·32 (5·83)	54·50 (6·64)	52·85 (6·42)	**0·01**	54·98 (6·05)	53·29 (6·23)	**<0·001**
GEMS score (categorical)							**0·02**			**<0·001**
Low (24-40)	16 (1·62)	8 (2·08)	5 (2·16)	0 (0)	2 (6·25)	1 (1·12)		6 (0·99)	10 (2·60)	
Medium (41-56)	597 (60·36)	241 (62·60)	139 (60·17)	141 (55·95)	15 (46·88)	61 (68·54)		341 (56·36)	256 (66·67)	
High (57-72)	376 (38·02)	136 (35·32)	87 (37·66)	111 (44·05)	15 (46·88)	27 (30·34)		258 (42·64)	118 (30·73)	
Women's age, years	24·07 (2·94)	23·31 (2·95)	24·02 (2·83)	24·64 (2·99)	24·22 (2·59)	23·84 (2·67)	**<0·001**	24·40 (2·86)	23·56 (2·99)	**<0·001**
Men's age, years	29·61 (3·68)	28·92 (3·75)	30·00 (3·31)	30·31 (3·98)	29·44 (2·07)	29·67 (2·74)	**<0·001**	29·80 (3·60)	29·31 (3·81)	**0·04**
		**n (%)**	**n (%)**	**n (%)**	**n (%)**	**n (%)**		**n (%)**	**n (%)**	
Women's education							0·30			0·57
No education or Primary	144 (14·56)	67 (46·53)	31 (21·53)	32 (22·22)	5 (3·47)	9 (6·25)		85 (59·03)	59 (40·97)	
Secondary or higher	845 (85·44)	318 (37·63)	200 (23·67)	220 (26·04)	27 (3·20)	80 (9·47)		520 (61·54)	325 (38·46)	
Men's Education							0·52			0·08
No education or Primary	138 (13·95)	57 (41·30)	36 (26·09)	28 (20·29)	6 (4·35)	11 (7·97)		75 (54·35)	63 (45·65)	
Secondary or higher	851 (86·05)	328 (38·54)	195 (22·91)	224 (26·32)	26 (3·06)	78 (9·17)		530 (62·28)	321 (37·72)	
Caste							**0·003**			0·32
General	672 (67·95)	242 (36·01)	165 (24·55)	167 (24·85)	27 (4·02)	71 (10·57)		404 (60·12)	268 (39·88)	
SC/ST/OBC	317 (32·05)	143 (45·11)	66 (20·82)	85 (26·81)	5 (1·58)	18 (5·68)		201 (63·41)	116 (36·59)	
Below Poverty Line card holder							0·38			0·86
Yes	244 (24·70)	107 (43·85)	51 (20·90)	59 (24·18)	9 (3·69)	18 (7·38)		456 (61·29)	288 (38·71)	
No	744 (75·30)	278 (37·37)	180 (24·19)	192 (25·81)	23 (3·09)	71 (9·54)		148 (60·66)	96 (39·34)	
Parity							**<0·001**			**<0·001**
0	104 (10·52)	73 (70·19)	17 (16·35)	14 (13·46)	0	0		38 (36·46)	66 (63·46)	
1	552 (55·81)	203 (36·78)	132 (23·91)	146 (26·45)	15 (2·72)	56 (10·14)		332 (60·14)	220 (39·86)	
2-4	333 (33·67)	109 (32·73)	82 (24·62)	92 (27·63)	17 (5·11)	33 (9·91)		235 (70·57)	98 (29·43)	
Any IPV (physical, sexual, or emotional)							0·11			0·14
Yes	213 (21·54)	76 (35·68)	60 (28·17)	47 (22·07)	5 (2·35)	25 (11·74)		121 (56·81)	92 (43·19)	
No	776 (78·46)	309 (39·82)	171 (22·04)	205 (26·42)	27 (3·48)	64 (8·25)		484 (62·37)	292 (37·63)	

*Note:* Excluded 212 couples from 1201 where either wife was pregnant (n = 199) or using an uncommon FP type (n = 4, all using injectable contraception), or where husband was missing GEMS score (n = 9). No husbands were missing responses for FP discussion with wife. *P* values are calculated from chi^2^ test for categorical and t-test or ANOVA for continuous variables.

Abbreviations: GEMS: Gender Equitable Men Scale, IPV: Intimate Partner Violence, IUD: Intrauterine Device, OBC: Other Backward Class, SC: Scheduled Caste, SD: Standard Deviation, ST: Scheduled Tribe.

### 3.2 Bivariate comparisons of outcomes by gender equitable attitudes score

Men's attitudes towards gender-equitable norms scores differed significantly by type of FP method used (*p* = 0·01); average GEMS score was highest (most equitable) among condom users (mean GEMS score 55·3). Average GEMS score was also significantly higher (more equitable) among those who discussed vs. those who did not discuss FP in the past three months with their wife (55·0 vs 53·3, *p* < 0·001).

### 3.3 Bivariate comparisons of outcomes and gender equitable attitudes score by sociodemographic characteristics

There were statistically significant differences in FP method use by women's age, men's age, women's caste, and women's parity. Condom users were the oldest group on average (24·6 for women, 30·3 for men, *p* < 0.001), while non-users of FP methods were the youngest group on average (23·3 for women, 28·9 for men p < 0.001). Women from Scheduled Tribes, Scheduled Castes, or Other Backwards Classes were more likely than those not in such classes to use no FP method (45% vs 36%, *p* = 0·003). Nulliparous women were much more likely to be using no FP method than multiparous women (70% vs 33%, *p* < 0·001). Similarly, there were statistically significant differences in recent FP discussion by women's age, men's age, and women's parity. Couples who had recently discussed FP were older on average (24·4 vs 23·6 years for women *p* < 0·001; 29·8 vs 29·3 years for men *p* = 0·04). Nulliparous women were less likely than multiparous women to have recently had a discussion about FP (36% vs 71%, p < 0·001). Most sociodemographic characteristics were significantly associated with GEMS score (see Table 2); age and education were positively associated with GEMS score (more equitable attitudes), while higher parity, BPL card ownership, and IPV history were negatively associated with GEMS score (less equitable attitudes).

**Table 2:** Unadjusted linear regression results indicating associations of socio-demographics with men's GEMS score among married couples in baseline sample from CHARM2 in rural Maharashtra, India (*N* = 989).

Socio-demographics	Unadjusted	
	*β Coef. (95% CI)*	*p value*
Men's age	0·14 (0·04, 0·24)	0·008
Women's age	0·23 (0·10, 0·36)	0·001
Couples' age difference	-0·01 (−0·13, 0·12)	0·929
Men's education		
No education	ref	ref
Secondary or high school	3·77 (2·68, 4·86)	<0·001
Women's education		
No education	ref	ref
Secondary or high school	1·84 (0·76, 2·93)	0·001
Caste		
None/Other	ref	ref
SC/ST/OBC	-0·09 (−0·92, 0·73)	0·826
Below Poverty Line card holder		
No	ref	ref
Yes	-1·44 (−2·33, − 0·55)	0·002
Parity		
None	-0·31 (−0·98, 1·60)	0·640
1	ref	ref
2+	-1·01 (−1·85, −0·18)	0·018
Any IPV		
No	ref	ref
Yes	-1·80 (−2·73, −0·86)	<0·001

Abbreviations: CI: Confidence Interval, GEMS: Gender Equitable Men Scale, IPV: Intimate Partner Violence, OBC: Other Backward Class, SC: Scheduled Caste, ST: Scheduled Tribe.

### 3.4 Unadjusted and adjusted regression analyses

Unadjusted multinomial logistic regression showed that with each point increase in GEMS score (e.g. more gender-equitable attitude) among men, there was a 4% increase in the relative risk of condom use compared to no use of FP (RRR 1.04, 95%CI 1·01-1·06, *p* = 0·01) (see Table 3). Adjustment for sociodemographic characteristics and IPV slightly attenuated these effects (ARRR = 1·03, 95%CI 1·00-1·06, p = 0·07). There was no significant association between GEMS score and use of other types of FP methods. Unadjusted logistic regression showed that higher GEMS score among men was significantly associated with greater odds of spousal discussion of family planning in the past three months (OR = 1·05, 95%CI 1·03-1·07, *p* < 0·001). Adjustment for sociodemographic characteristics and IPV did not modify these effects (AOR = 1·05, 95%CI 1·03-1·07, p < 0·001). Results from our post-hoc exploration of GEMS and IPV suggest that higher GEMS score among men was associated with slightly but statistically significantly lower odds of IPV (e.g. any IPV AOR 0·96, 0·93 − 0·98) (see Appendix Table 2). In seeing that GEMS score was only significantly associated with condom use, we hypothesized that this association might be explained at least partially by increased communication, and thus we conducted a final additional post-hoc analyses to explore this issue. We examined the extent to which inclusion of marital communication mediated the observed effects between GEMS score and condom use. GEMS score was no longer significantly associated with condom use in adjusted multinomial logistic regression models controlling for communication (AOR 1·02, 95% CI 0·99 − 1·05), suggesting at least partial mediation (results not shown).

**Table 3:** Unadjusted and adjusted regression between men's GEMS score and use of types of FP methods (multinomial logistic regression), and FP discussion (binary logistic regression) among married couples in rural Maharashtra, India (N = 989).

	Unadjusted		Adjusted	
Type of family planning method used				
	*RRR (95% CI)*	*p value*	*ARRR (95% CI)*	*p value*
None	Ref		Ref	
Traditional	1·00 (0·98-1·03)	0·62	1·01 (0·98-1·04)	0·56
Condoms	**1·04 (1·01-1·06)**	**0·01**	**1·03 (1·00-1·06)**	**0·07**
Pills	1·01 (0·95-1·07)	0·67	1·02 (0·96-1·09)	0·44
IUD	0·97 (0·93-1·01)	0·11	0·97 (0·93-1·01)	0·16
Family planning discussion with wife				
	*OR (95% CI)*	*p value*	*AOR (95% CI)*	*p value*
No	Ref		Ref	
Yes	**1·05 (1·03-1·07)**	**<0·001**	**1·05 (1·03-1·07)**	**<0·001**

*Note:* Adjusted for men's age, women's age, men's education, women's education, caste, Below Poverty Line card holder, parity, any intimate partner violence.

Abbreviations: AOR: Adjusted Odds Ratio, ARRR: Adjusted Relative Risk Ratio CI: Confidence Interval, GEMS: Gender Equitable Men Scale, IUD: Intrauterine Device, OR: Odds Ratio, RRR: Relative Risk Ratio;

## 4 Discussion

Our study sought to understand the role of men's gender-equitable ideologies, as measured by GEMS score, on family planning in India with a sample of married men rural Maharahstra. We found that men who hold more equitable gender ideologies are significantly more likely to discuss contraceptive use and use condoms with their wives. Findings related to contraceptive communication are consistent with prior research from other national contexts [[10], [11], [12], [13]], findings related to condom use are consistent with prior research from India [26,28]. We did not find that men's gender ideologies were associated with other forms of contraceptives.

It may be the case that gender role ideologies may support male involvement in contraceptive decision-making, communication, and use, but that this male engagement is tied primarily to male-controlled contraceptives. In the absence of more effective reversible contraceptives that men can use, we see male engagement primarily in a less effective contraceptive (condoms). These findings reinforce ongoing calls for better male contraceptive options, and at the same time suggest that gender-equity focused programs to engage males in FP may best support *effective* FP use by including female partners as well. Such findings are consistent with our Can-Act-Resist framework, which centralizes women's agency for reproductive decision-making even while supporting engagement of men in family planning [19].

These findings are consistent with prior research that suggests more equitable gender norms are linked to modern contraceptive use, and in particular, condom use. While several studies have shown that men's attitudes about gender equity are associated with condom use to prevent HIV [33,34], Mishra et al. also explored whether the relationship held true outside the HIV context and found that among FP users, men with more gender-equitable attitudes were more likely to use condoms [35]. A study of a gender-equity focused family planning program engaging males in rural India also found that the significant effects of the program on contraceptive use were primarily explained by significant increases in condom use [36]. Research focused on men's gender attitudes and female-controlled modern methods indicate no significant association between these [35].

We also found that men's gender-equitable attitudes were positively associated with spousal communication about FP. However, communication alone does not ensure equitable FP decision-making. For example, studies in Ethiopia and Tanzania have found that even when FP-related communication is taking place within a relationship, male partners were the final decision makers with regards to whether and how to use family planning [40,41]. In such settings, male engagement that avoids reinforcing dominant gender roles in decision-making is needed [42], and programs which directly address gender attitudes and norms may be one way to achieve this aim.

Post-hoc analysis also suggests that communication at least partially mediates the relationship between men's masculinity ideologies and condom use. This suggests that FP discussion may lead to condom use. However, our analysis does not provide insight into the full FP decision-making process between members of a couple, including whether only one or both partners wanted to use condoms, and who had final say on the decision to use condoms in the case of disagreement. As aligned with our framework, sexual and reproductive efforts should maintain women as central – and in fact the priority over male partners – with regard to use of contraceptives and fertility decision-making, as they ultimately bear the burdens and responsibilities of reproduction.

These data do not allow us to understand the full context of contraceptive use, nor the full mechanisms through which masculinity ideologies may affect contraceptive use, beyond contraceptive communication. These norms may be a marker for less traditional ideologies generally, and a greater openness to new ideas, such as male responsibility via condom use. But they do not necessarily align with recognition of the importance of women being the primary decision-makers of choice and timing regarding reproduction. Inclusion of qualitative data and mixed methods analyses to understand the dynamics of contraceptive use in marriage may help offer insight beyond what can be provided in this quantitative study.

While it is ultimately unsurprising that men's attitudes are more strongly related to use of methods they control themselves than to methods controlled by their partner, this finding has programmatic implications for the use of gender-transformative approaches with the goal of uptake of highly effective modern contraceptives. More specifically, the findings support male engagement in family planning and addressing traditional male gender role norms supportive of male dominance, as these likely affect male support and use of contraceptives in rural India.

This study contributes to the current understanding of gender equity norms and contraceptive use in rural India, but must be viewed in light of several limitations. The study is limited to a sample of young husbands and non-sterilized wives in a single district of rural Maharashtra, and thus, generalizability of study findings may be more limited to rural India or other similar lower resource settings. In this context, condoms and pills are available at no or low cost from the public health system, and findings from this study may not be reflective of places where cost burdens can prohibit use of these contraceptives. This study is cross-sectional; while there is theoretical justification as to why more equitable gender norms would lead to more desirable FP outcomes [19], we cannot presume causality from these findings. Small cell sizes by type of contraceptive may also be a concern; while no associations between gender-equitable norms and contraceptive pill use were found, our findings are limited by a small number (*n* = 32) of pill users and further research on the relationship between attitudes and pill use is warranted.

## 5 Conclusion

This study contributes to our understanding of the association between men's gender attitudes and contraceptive use and communication. Findings extend prior work documenting the association between these attitudes and condom use by also showing their association with contraceptive communication, but not with other types of contraceptives. Further, we see that the association between more equitable masculinities ideologies and condom use is mediated by FP communication with one's partner. Less clear is whether this communication results in male controlled or couple's shared decision-making regarding condom use. These findings suggest that gender-equity-focused FP programs which aim to increase effective method utilization and which include men must consider how to provide these programs in ways that support both male engagement and women's control of more effective method use. Such findings reinforce the value of couple-level and gender-synchronized interventions to ensure that women's engagement and control remain central in gender-transformative FP programming [9,43]. Additionally, development of more effective reversible contraceptives for men would allow males who are engaged in FP decision-making to be able to effectively take responsibility for contraceptive use when it is the couple's goal. Ultimately, men's gender-equitable attitudes may contribute to discussion and utilization of FP, but full realization of these outcomes may be hindered by availability of male-controlled methods and lack of female agency in contraceptive decision-making and control.

## Role of the funding source

This work was supported by the 10.13039/100000002National Institutes of Health [Grant number 5R01HD084453-01A1] and the 10.13039/100000865Bill & Melinda Gates Foundation, Seattle, WA [grant number INV-002967]. The funding source had no involvement in the study design; in the collection, analysis, or interpretation of data; in the writing of the report; or in the decision to submit the paper for publication.

## CRediT authorship contribution statement

**Mohan Ghule:** Conceptualization, Project administration, Supervision, Writing – original draft, Writing – review & editing. **Anvita Dixit:** Formal analysis, Project administration, Writing – original draft. **Nicole E. Johns:** Data curation, Methodology, Project administration, Writing – original draft, Writing – review & editing. **Madhusudana Battala:** Conceptualization, Writing – review & editing. **Shahina Begum:** Conceptualization, Project administration, Supervision, Writing – review & editing. **Sarah Averbach:** Writing – review & editing. **Jay G. Silverman:** Writing – review & editing. **Niranjan Saggurti:** Writing – review & editing. **Anita Raj:** Conceptualization, Funding acquisition, Supervision, Writing – original draft, Writing – review & editing.

## Declaration of competing interest

The authors declare no conflicts of interest.

## Data Availability

Data used in this study is available upon reasonable request to the corresponding authors.

## Appendix A Supplementary data

Appendix Table 1: Gender Equitable Men Scale items Image 1
