# Supplementary material for: Examining the association between men's gender equitable attitudes and contraceptive outcomes in rural Maharashtra, India
Source: Dialogues Health. 2024 Jun;4:100168. doi: 10.1016/j.dialog.2024.100168 (PMC10953923; doi:10.1016/j.dialog.2024.100168)
Supplement: Supplementary file 1 — Appendix Table 1: Gender Equitable Men Scale items [file mmc1.pdf]

Appendix Table 1: Gender Equitable Men Scale items

Item	Text	Coding
1	It is the man who decides what type of sex to have.	Standard
2	A woman's most important role is to take care of her home and cook for her family.	Standard
3	Men need sex more than women do.	Standard
4	You don't talk about sex, you just do it.	Standard
5	Women who carry condoms on them are "easy."	Standard
6	A man needs other women, even if things with his wife are fine.	Standard
7	There are times when a woman deserves to be beaten.	Standard
8	Changing diapers, giving the kids a bath, and feeding the kids are the mother's responsibility.	Standard
9	It is a woman's responsibility to avoid getting pregnant.	Standard
10	A man should have the final word about decisions in his home.	Standard
11	Men are always ready to have sex.	Standard
12	A woman should tolerate violence in order to keep her family together.	Standard
13	If a woman cheats on a man, it is okay for him to hit her.	Standard
14	If someone insults me, I will defend my reputation, with force if I have to.	Standard
15	I would be outraged if my wife asked me to use a condom.	Standard
16	It is okay for a man to hit his wife if she won't have sex with him.	Standard
17	I would never have a gay friend.	Standard
18	A couple should decide together if they want to have children.	Reverse
19	In my opinion, a woman can suggest using condoms just like a man can.	Reverse
20	If a guy gets a woman pregnant, the child is the responsibility of both.	Reverse
21	A man should know what his partner likes during sex.	Reverse
22	It is important that a father is present in the lives of his children, even if he is no longer with the mother.	Reverse
23	A man and a woman should decide together what type of contraceptive to use.	Reverse
24	It is important to have a male friend that you can talk about your problems with.	Reverse

Appendix Table 2: Unadjusted and adjusted logistic regression between men's attitudes towards gender norms (GEMS score) and any intimate partner violence among married couples in rural Maharashtra, India (N=989).

	**Unadjusted**		**Adjusted**	
**Any IPV**				
	OR (95% CI)	*p value*	*AOR (95% CI)*	*p value*
**No**	ref		ref	
**Yes**	**0.95 (0.93-0.98)**	**<0.001**	**0.96 (0.93-0.98)**	**0.001**
**Physical IPV**				
**No**	ref		ref	
**Yes**	**0.95 (0.92-0.98**	**0.004**	**0.96 (0.93-0.99)**	**0.016**
**Sexual IPV**				
**No**	ref		ref	
**Yes**	**0.93 (0.88-0.99)**	**0.029**	0.94 (0.88-1.00)	0.065
**Emotional IPV**				
**No**	ref		ref	
**Yes**	**0.95 (0.93-0.98**	**0.001**	**0.96 (00.93-0.99)**	**0.004**

Note: Adjusted for men's age, women's age, men's education, women's education, caste, Below Poverty Line card holder, parity.
